# The Red Cross Society in Yorkshire

**Published:** 1912-09-21

**Authors:** 


### The Red Cross Society in Yorkshire.

### PARADE AND CUP COMPETITION.

Last Saturday a field-day and parade of the North Riding Branch of the Red Cross Society was held at Redcar Racecourse. The twenty-six voluntary aid detachments which were on parade were inspected by General Sir Ian Hamilton, with whom were Surgeon-General Kenny and the Lord Lieutenant of the North Riding, Sir Hugh Bell. The parade was followed by an examination of the detachments, the examiners including both Army and civilian doctors, and the teams chosen then competed for the two, men's and women's, challenge cups, which had been presented by Sir T. Willans Nussey, the county director, and Lady Bell respectively. The winning teams were those of Thirsk and Richmond.

In presenting the cups to the successful teams Sir Ian Hamilton emphasised the progress of antiseptic surgery and its value in modern warfare, pointing out that properly staffed and equipped detachments could do more for the wounded to-day than at any previous time in the history of Red Cross work. A large gathering, representative of all parts of the North Riding, witnessed the parade, and among the spectators was the Postmaster-General.

